# Associations between general and specific mental health conditions in young adulthood and cardiometabolic complications in middle adulthood: A 40-year longitudinal familial coaggregation study of 672 823 Swedish individuals

**DOI:** 10.1192/j.eurpsy.2023.230

**Published:** 2023-07-19

**Authors:** C. Chen, Z. Chang, R. Kuja-Halkola, B. M. D’Onofrio, H. Larsson, P. Andell, P. Lichtenstein, E. Pettersson

**Affiliations:** 1 Karolinska Institutet, Stockholm, Sweden; 2 Indiana University, Bloomington, United States; 3 Örebro University, Örebro; 4 Karolinska University Hospital, Stockholm, Sweden

## Abstract

**Introduction:**

Most mental disorders, when examined individually, are associated with an increased risk of cardiometabolic complications. However, these associations might be attributed to a general liability toward psychopathology or confounded by unmeasured familial factors.

**Objectives:**

To examine whether the associations between psychiatric diagnoses and increased risk of cardiometabolic complications are attributable to a general liability toward psychopathology, or confounded by unmeasured familial factors.

**Methods:**

We conducted a cohort study in Sweden and identified all individuals and their siblings born in Sweden 1955-1962 with follow-up through 2013. After excluding individuals who died or emigrated before 1987, the final sample consisted 672 823 individuals. We extracted ICD-coded diagnoses (recorded 1973-1987) for ten psychiatric conditions and criminal convictions when participants were aged 18-25 years, and ICD-coded diagnoses (recorded 1987-2013) for five cardiometabolic complications (obesity, hypertensive diseases, hyperlipidemia, type 2 diabetes mellitus, and cardiovascular diseases) when the participants were 51-58 years old. Logistic regression models were used to estimate the bivariate associations between psychiatric conditions or criminal convictions and cardiometabolic complications in individuals. A general factor model was used to identify general, internalizing, externalizing, and psychotic factors based on the psychiatric conditions and criminal convictions. We then regressed the cardiometabolic complications on the latent general factor and three uncorrelated specific factors within a structural equation modeling framework in individuals and across sibling pairs.

**Results:**

Each psychiatric conditions significantly increased the risk of cardiometabolic complications; however, most of these associations were attributable to the general factor of psychopathology, rather than to specific psychiatric conditions. There were no or only small associations between individuals’ general psychopathology and their siblings’ cardiometabolic complications, suggesting that the associations were not attributable to genetic or environmental confounding factors shared within families. The same pattern was evident for the specific internalizing and psychotic factors.

**Conclusions:**

Individuals with mental disorders in early life had an increased long term risk of cardiometabolic complications, which appeared attributable to a general liability toward psychopathology. Sibling analyses suggested that the elevated risk could not be attributed to confounds shared within families.This highlights the importance of transdiagnostic and lifestyle based interventions to reduce the risk of cardiometabolic complications, particularly in patients with several mental disorders.

**Disclosure of Interest:**

None Declared

